# Lensless ultrafast optical imaging

**DOI:** 10.1038/s41377-022-00789-6

**Published:** 2022-04-18

**Authors:** Jian Zhao, Mingsheng Li

**Affiliations:** Department of Electrical and Computer Engineering, Boston University, Boston, MA 02215 USA

**Keywords:** Optics and photonics, Electronics, photonics and device physics

## Abstract

Lensless single-shot ultrafast optical imaging is realized by integrating an acousto-optic programmable dispersive filter with spectrally filtered sequentially time all-optical mapping photography, which enables independent control of frame rate, frame intensity, and exposure time with a simple system design.

High-speed transient dynamic phenomena widely exist and affect nearly all areas of human society, such as industry, defense, biomedicine, and scientific research^1,2^. Visualization of the high-speed transient dynamic process plays a pivotal role in discovering, understanding, and applying the underlying laws^3–7^. Yet, capturing and recording blur-free images of the transient dynamics requires high temporal resolution with balanced spatial resolution, which imposes demanding conditions on imaging acquisition techniques. The first recorded high-speed photography by William Henry Fox Talbot dates back to 1851 when a single camera was used to photograph a page of Times newspaper fastened on a spinning drum illuminated by a fast spark light source^8^. Fox Talbot demonstrated a clear print of the page, the motion of which was effectively frozen. This demonstration stands for the born of high-speed photography. Instead of actively modulating the light source, Eadweard Muybridge performed another famous high-speed photography experiment by passively recording the dynamics of a galloping horse using an array of cameras in 1878 (ref. ^9^). Interestingly, the different solutions from these early attempts correspond to two important domains of modern ultrafast optical imaging research, i.e., active detection and passive detection.

Nowadays, the motion of a spinning drum or a galloping horse can be easily recorded by a modern electronic sensor, such as the charge-coupled device (CCD) camera or complementary metal-oxide-semiconductor (CMOS) camera. However, the frontier scientific research often investigates ultrafast dynamical processes hard to be captured by CCD or CMOS, such as flow cytometry of biological cells^10^, laser pulse propagation^11^, and phonon dynamics in crystal^12^. To visualize these ultrafast dynamics, it requires acquisition speed from ~100 million fps (Mfps) up to ~1 trillion fps (Tfps). Unfortunately, it is quite challenging to go beyond ~10 Mfps for CCD and CMOS cameras due to the limitation of the on-chip storage and electronic readout speed^13^. Currently, the popular time-resolved pump-probe imaging method^14,15^ typically provides frame intervals on the order of ~100 fs, enabling the capture of the ultrafast phenomena through repeated measurements. Despite the high temporal resolution, the pump-probe method is difficult to detect non-repetitive or difficult-to-reproduce events, such as irreversible crystalline chemical reactions, quantum-mechanical processes, and optical rogue waves.

To circumvent the technical hurdles from the pump-probe method, various single-shot ultrafast optical imaging solutions have been developed in recent years^3–7,16^. These technologies enable the visualization of non-repetitive ultrafast dynamic processes in real time with an imaging speed of 100 Mfps or higher. Similar to the pioneer high-speed photography experiments in the 1900s, these single-shot ultrafast optical imaging methods can be generally categorized as active detection methods and passive detection methods^4^. Active-detection methods probe the ultrafast dynamics through modulation of the ultrafast optical pulses, while passive-detection methods directly image the dynamics or perform computational reconstruction without active illumination. Among different active-detection solutions, the sequentially time all-optical mapping photography (STAMP)^12^ reaches a record high frame rate of ~1 Tfps. STAMP temporally shapes the ultrashort pulse into a pulse train containing several daughter pulses with different center wavelengths. These daughter pulses successively detect the dynamics of the targets and are further spatially separated and projected onto the image sensor to produce a motion picture. Despite its superior imaging performance, STAMP requires a complex and bulky optical system consisting of a pulse stretcher and a pulse shaper. A later improved design, termed spectrally filtered STAMP (SF-STAMP)^17^, simplified the STAMP by abandoning the pulse shaper and using diffractive optical elements together with a tilted bandpass filter. In spite of the improvements, SF-STAMP still relies on a complex pulse stretcher that contains hollow-core optical fiber and glass rods, which is not user-friendly and exhibits low flexibility regarding the control of frame rate and exposure time.

Recently, Touil et al.^18^ demonstrated a novel lensless solution to address the issues of system complexity and inflexibility by integrating the acousto-optic programmable dispersive filter (AOPDF) with the SF-STAMP scheme. The principle of this new method is illustrated in Fig. 1. An ultrashort pulse is sent into the AOPDF’s crystal, where the optical wave interacts with the radio-frequency-driven acoustic control wave through a frequency mixing process. By tailoring the acoustic control wave, the phase and amplitude of the output optical spectrum can be precisely shaped. The pulse shaping process in the AOPDF generates a pulse train consisting of quasi-equalized daughter pulses shifted temporally and with different center wavelengths. Here, an individual daughter pulse works as a single frame that encodes the imaging information of the object. The width of each daughter pulse corresponds to the exposure time of ~723 fs, while the time between neighboring pulses (~2 ps) defines the frame rate. As shown in Fig. 1, those five temporally shifted daughter pulses interact with the ultrafast changing object sequentially to encode imaging information from the consecutive transient dynamics. Then, daughter pulses in the pulse train are further spatially separated based on their different center wavelengths through a combination of a diffractive optical element and a tilted spectral filter. After the filtering process, those daughter pulses are projected onto different areas of the same CCD camera sensor. The ultrafast imaging capabilities and the imaging flexibilities of this AOPDF-based method are validated by visualizing the optical Kerr gate process within the picosecond regime and capturing the laser-induced ablation dynamics in the nanosecond regime. Such measurements under different time scales are achieved using external optical delay lines. Single-shot ultrafast optical phase imaging using digital in-line holography is also demonstrated by visualizing laser-induced air breakdown dynamics in three-dimensional space.

**Fig. 1:**
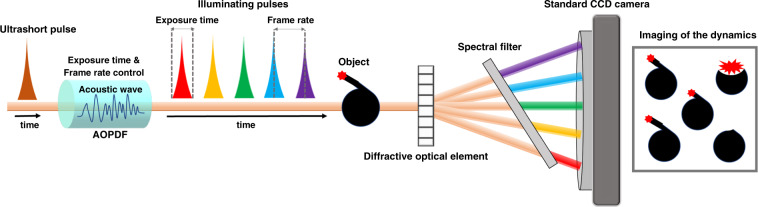
Principle of the lensless ultrafast optical imaging. Acousto-optic programmable dispersive filter (AOPDF) performs pulse shaping on an ultrashort pulse, generating a pulse train. Each daughter pulse within the pulse train works a single frame encoding the imaging information and is spatially separated from other daughter pulses. All the daughter pulses are finally projected onto different areas of the camera sensor

The abovementioned tremendous progress is mainly attributed to the applications of the AOPDF in the SF-STAMP. As a compact and turn-key device, the AOPDF not just maximizes the system’s simplicity but also provides a high degree of freedom regarding the optical pulse parameters tuning through a user-friendly lensless design. Specifically speaking, the AOPDF-based ultrafast optical imaging solution is free from conventional bulky and complex pulse shaping configuration as well as avoids the usage of complicated computational algorithms. This method enables independent control of frame rate, exposure time, and frame intensity. It also achieves a simple transition between different detection time scales. Compared to other state-of-the-art solutions, AOPDF-based SF-STAMP has a great potential to be easily deployed outside the laboratory environment. Overall, AOPDF-based solution achieves significant progress in breaking the barrier of complexity and inflexibility in the field of single-shot ultrafast optical imaging.

Finally, this work sheds light on revolutionizing other active-detection technologies and paves the way for many new applications. The novel concept of boosting the system with a simple integrated optical device would inspire innovations on different active single-shot ultrafast optical imaging solutions, generating various effective and user-friendly imaging platforms. Meanwhile, the application of the AOPDF-based method could be extended to many other areas by further refinement and revision, such as biomedicine. For example, the unique ultrafast optical phase imaging capabilities could be adapted to a novel microscopy platform for observing biological phenomena. All the above-mentioned aspects could be the future directions of this area, and we expect it to have a more significant impact on both fundamental research and practical applications.
